# Supplementary material for: Connexin 26 and Connexin 43 in Canine Mammary Carcinoma
Source: Vet Sci. 2019 Dec 9;6(4):101. doi: 10.3390/vetsci6040101 (PMC6958330; doi:10.3390/vetsci6040101)
Supplement: Supplementary file 1 [file vetsci-06-00101-s001.pdf]

veterinary sciences

MDPI

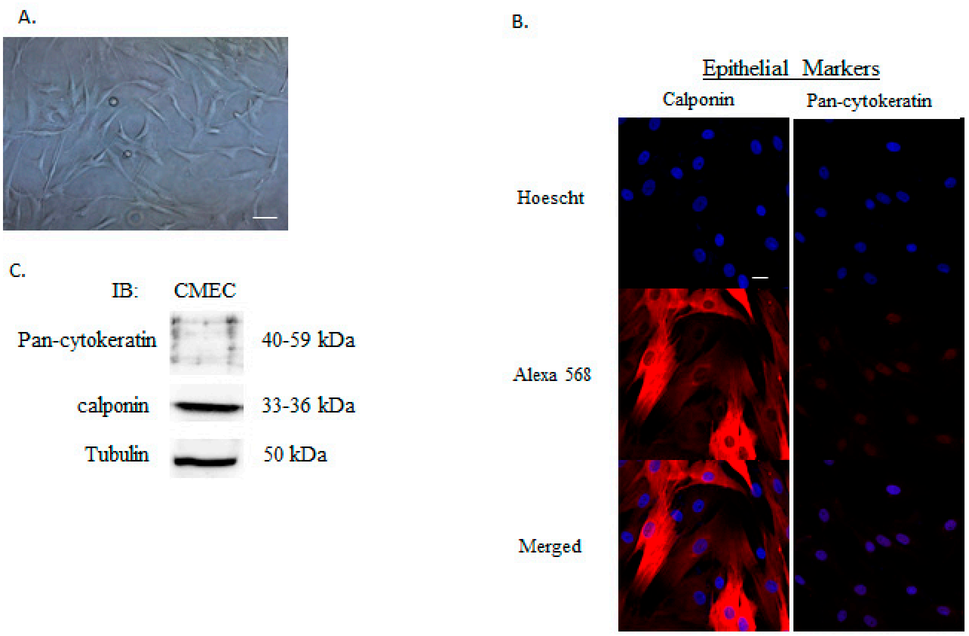

Figure S1. Charaterization of Canine Mammary Epithelial Cells. Micrograph of CMEC in cell culture at 20X (**A**). Cells were screened for epithelial markers using immunocytochemistry (**B**) and Western blot (**C**). The bar is equivalent to 20 µm.
